# Projections of the Current and Future Disease Burden of Hepatitis C Virus Infection in Malaysia

**DOI:** 10.1371/journal.pone.0128091

**Published:** 2015-06-04

**Authors:** Scott A. McDonald, Maznah Dahlui, Rosmawati Mohamed, Herlianna Naning, Fatiha Hana Shabaruddin, Adeeba Kamarulzaman

**Affiliations:** 1 Centre of Excellence for Research in AIDS (CERiA), University of Malaya, Kuala Lumpur, Malaysia; 2 Centre for Infectious Disease Control, National Institute for Public Health and the Environment, Bilthoven, The Netherlands; 3 School of Health & Life Sciences, Glasgow Caledonian University, Glasgow, Scotland; 4 Julius Centre, Faculty of Medicine, University of Malaya, Kuala Lumpur, Malaysia; 5 Faculty of Medicine, University of Malaya, Kuala Lumpur, Malaysia; Centers for Disease Control and Prevention, UNITED STATES

## Abstract

**Background:**

The prevalence of hepatitis C virus (HCV) infection in Malaysia has been estimated at 2.5% of the adult population. Our objective, satisfying one of the directives of the WHO Framework for Global Action on Viral Hepatitis, was to forecast the HCV disease burden in Malaysia using modelling methods.

**Methods:**

An age-structured multi-state Markov model was developed to simulate the natural history of HCV infection. We tested three historical incidence scenarios that would give rise to the estimated prevalence in 2009, and calculated the incidence of cirrhosis, end-stage liver disease, and death, and disability-adjusted life-years (DALYs) under each scenario, to the year 2039. In the baseline scenario, current antiviral treatment levels were extended from 2014 to the end of the simulation period. To estimate the disease burden averted under current sustained virological response rates and treatment levels, the baseline scenario was compared to a counterfactual scenario in which no past or future treatment is assumed.

**Results:**

In the baseline scenario, the projected disease burden for the year 2039 is 94,900 DALYs/year (95% credible interval (CrI): 77,100 to 124,500), with 2,002 (95% CrI: 1340 to 3040) and 540 (95% CrI: 251 to 1,030) individuals predicted to develop decompensated cirrhosis and hepatocellular carcinoma, respectively, in that year. Although current treatment practice is estimated to avert a cumulative total of 2,200 deaths from DC or HCC, a cumulative total of 63,900 HCV-related deaths is projected by 2039.

**Conclusions:**

The HCV-related disease burden is already high and is forecast to rise steeply over the coming decades under current levels of antiviral treatment. Increased governmental resources to improve HCV screening and treatment rates and to reduce transmission are essential to address the high projected HCV disease burden in Malaysia.

## Introduction

Infection with the hepatitis C virus (HCV) is an important emerging public health issue for both developed and developing nations; an estimated 185 million people world-wide have been infected with HCV [1]. Southeast Asia has a high prevalence of viral hepatitis, and it is estimated that more than 11 million people in this region have HCV [2]. The WHO Framework for Global Action on Viral Hepatitis [3] encourages all countries to estimate their national disease burden due to viral hepatitis. Therefore, this research addresses the question: ‘What is the projected disease burden due to past and future infection with HCV in Malaysia’?

As a preliminary step to answering this question, multi-parameter evidence synthesis modelling methods [4], [5] were employed to estimate the number of living HCV antibody-positive (Ab+) persons in 2009 [6]. This approach combined all available relevant data sources—both direct and indirect—that inform the epidemiological parameters of interest. These parameters include the population prevalence of people who inject drugs (PWID), the prevalence of hepatitis C in PWID and in non-PWID risk groups, and the distribution over drug injecting and other modes of transmission (e.g., receipt of contaminated blood products, non-sterile medical injection, haemodialysis, occupational exposure, tattooing, sexual contact, perinatal infection). The evidence synthesis approach provides statistically robust estimates, and importantly correctly propagates the uncertainty in the component data sources to the parameters being estimated.

In the current paper, our aim was to combine these previously-derived prevalence estimates, with annual transition probabilities between HCV-related disease stages taken from the literature and assumptions about the historical HCV incidence time series, to estimate the current population-level disease burden due to HCV, and to forecast the future disease burden to the year 2039. We projected the incidence of cirrhosis, decompensated cirrhosis (DC), and hepatocellular carcinoma (HCC), and death from DC or HCC, as well as the composite disability-adjusted life-years (DALY) and years of life lost (YLL) measures. This research adds to the projection exercises recently conducted for Australia, England & Wales, USA, and other countries [7], [8], [9] (see [10] for a review of several recent national studies), and is one of the few HCV projection models implemented for low/middle-income countries [11].

## Methods

To estimate the current and future HCV-related disease burden, the following data sources are required: (i) the time-series of incident cases from the year when HCV began circulating to present, and projected into the future; (ii) the distribution of the age at acute infection; (iii) a natural history model of HCV disease, with progression probabilities from acute infection through severe sequelae and death. Regarding (i), we had almost no information on HCV-related outcomes, and so could not attempt to back-calculate the incidence time-series from HCC deaths, for example, as done in previous research [12]. The alternative was to implement and compare several different assumptions about the pattern **of** historical incidence via scenario analysis.

### Simulating historical and future incidence

The incidence time-series was simulated so that the modelled number of prevalent living Ab+ individuals in 2009, calculated separately for PWID and non-PWID risk groups, equalled the median estimated number of prevalent living HCV Ab+ persons derived via evidence synthesis (total = 453,700, 95% credible interval: 391,700 to 535,100; total PWID = 268,750; total non-PWID = 184,950) [6]. There are many possible patterns of historical incidence that could have produced these 2009 prevalence values; we have chosen to simulate a very simple transmission situation, given that there are no data available on HCV incidence in Malaysia. We then compared this baseline scenario (Scenario 1) with two other plausible historical incidence scenarios (see below).

The most significant impact on historical HCV incidence among PWID is likely attributable to Malaysia’s national harm reduction programme (methadone maintenance therapy (MMT) and needle/ syringe provision (NSP)), which has expanded rapidly since its inception in 2005. By June 2014, approximately 35,000 persons had registered for MMT services at public sector sites (Ministry of Health hospitals and clinics, prisons and National Anti-Drug Agency service centres), and a further 30,000 PWID had accessed MMT services at GP clinics [13]. In the same time period, approximately 73,000 PWID had registered to receive NSP services. [13]

**Fig 1:**
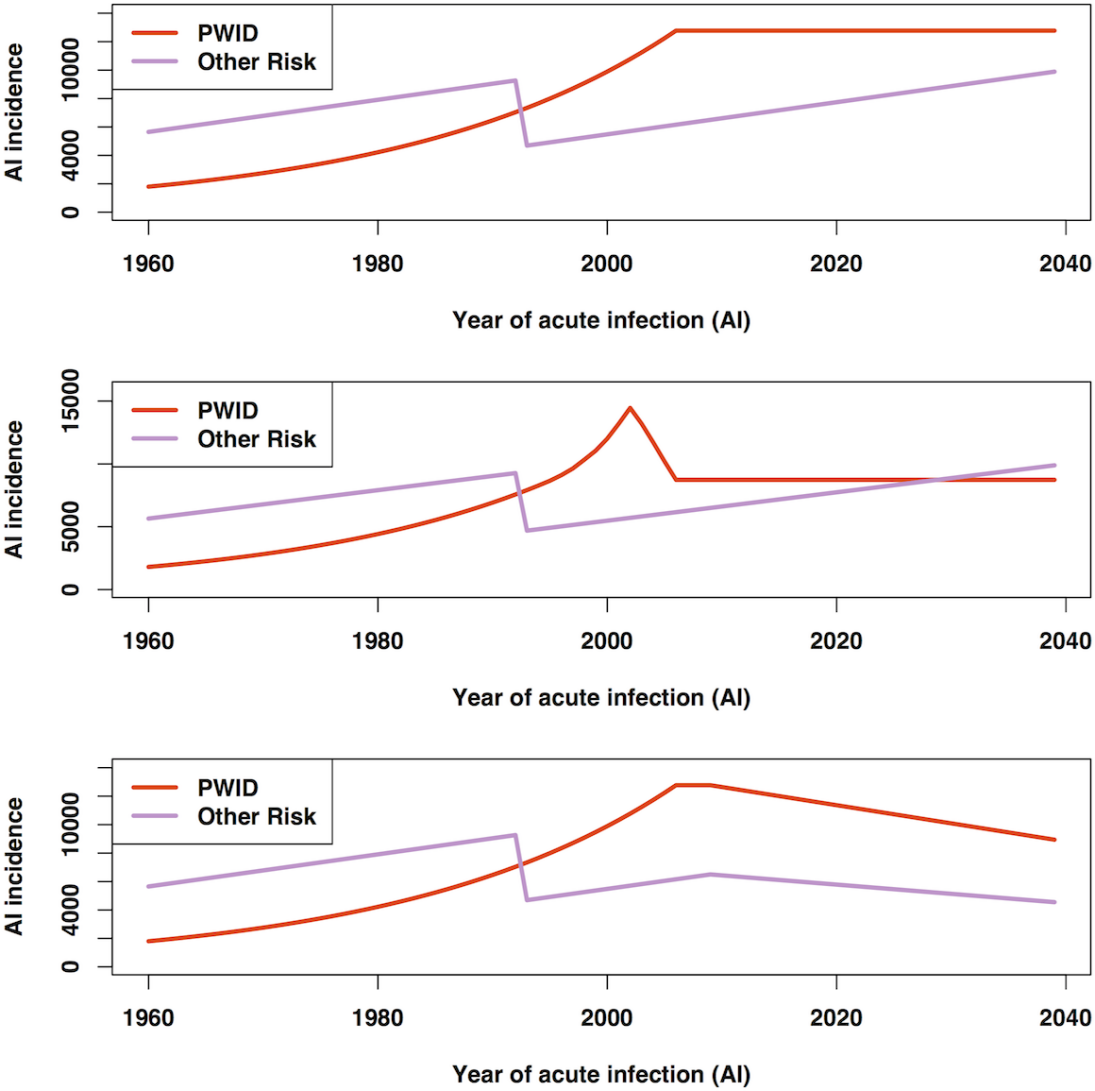
Patterns of acute incidence underlying Scenarios 1 to 3. The assumed historical HCV incidence time series for Scenarios 1 to 3 are shown separately for PWID and non-PWID risk groups (upper, centre, and lower panels, respectively).

In the baseline scenario, we assumed an exponential increase in HCV incidence among PWID, from an initial value of 1800 acute infected persons per year in 1960, which then levelled off in 2006. This stabilisation simulates the plausible impact on HCV transmission from harm reduction measures aimed at reducing HIV transmission among PWID (Fig 1). The parameter specifying the rate of increase in acute HCV incidence was adjusted so that the modelled number of living HCV Ab+ PWID in 2009 fitted the median estimated HCV prevalence among PWID in the same year. From 2006 onwards, annual incidence among PWID was assumed constant at the 2006 value (12,770 infections per year). For non-PWID modes of transmission, we assumed a linear rate of increase of 2% per year in the annual number of acute infections from 1960 through 1992, roughly consistent with the rate of population growth [14]. From 1993 onwards, we assumed a drop in annual incidence of 50% (to 4689 per year in 1993) to reflect the impact of blood donor screening for HCV antibody (Fig 1). This is the same assumption used by previous models [12], [15]. Given population growth since the beginning of the simulation period, the simulated increasing linear trend in the annual number of infected non-PWID corresponds to a relatively constant incidence rate over time.

In Scenario 2, incidence among PWID was assumed to follow the approximate shape of the HIV epidemic, in which a peak of new HIV diagnoses was reported for 2002 [16]. Scenario 3 was identical to Scenario 1, except that a potential effect of harm reduction initiatives on incidence in PWID and the impact of improved awareness and education on incidence in non-PWID was simulated, by assuming for both subpopulations that from 2010, the number of incident cases declined at a linear rate of 1% per year from 2009 levels.

The model-predicted total number of living HCV Ab+ persons in 2009 was 452,200, 452,000, and 452,300, for baseline Scenarios 1 through 3, respectively.

**Fig 2:**
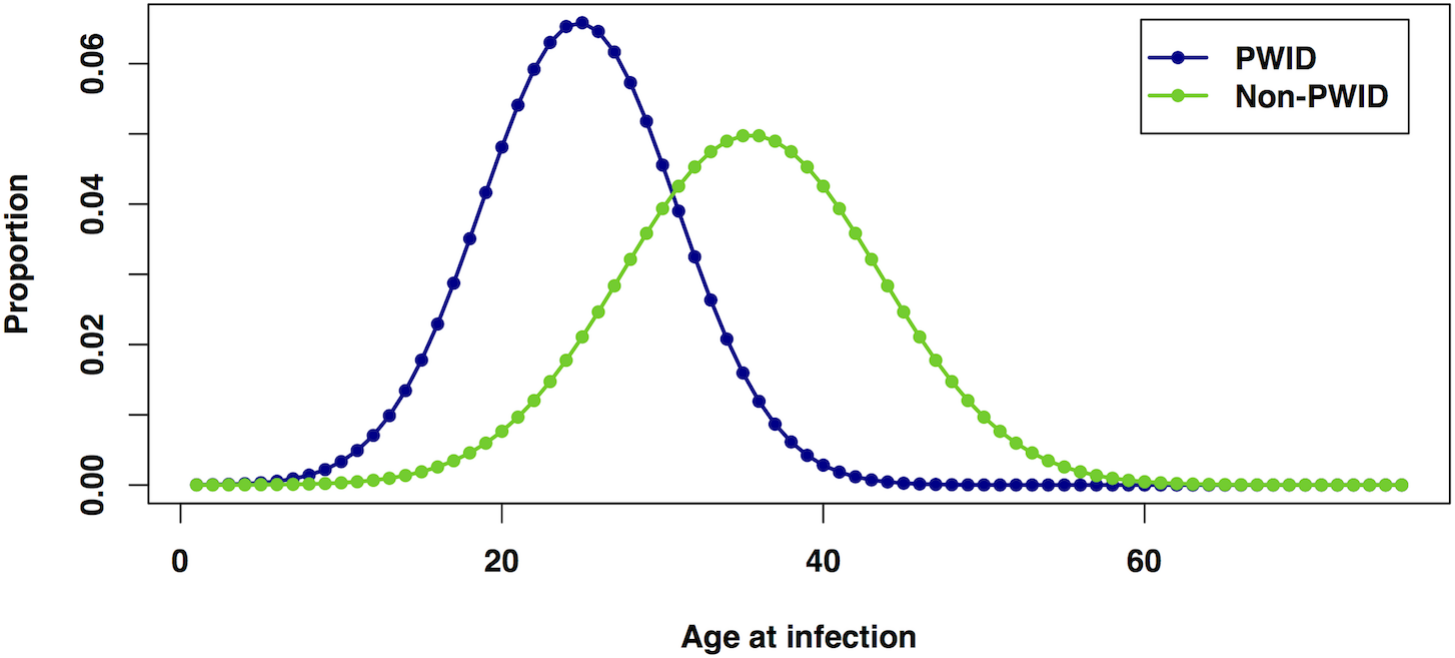
Distributions of age at acute infection, for PWID and non-PWID.

The distribution of age at acute infection was assumed to differ between PWID and non-PWID subpopulations (Fig 2). For PWID, the average reported age at first injection from a community-based study of injecting drug users [17] was used as a proxy for the average age of acute infection. Thus, a normal distribution with mean of 24.3 years was assumed, with a standard deviation set to the value from the same study (6.05 years). The average age at infection for non-PWID was assumed to be older compared with non-PWID; this was defined as a normal distribution with mean of 35 years and standard deviation of 8 years.

### Modelling disease progression

Simulating disease progression and projection of HCV-related disease outcomes was carried out using a multi-state Markov modelling approach, similar to previously published models [7], [8], [11], [18], [19], [20].

**Fig 3:**
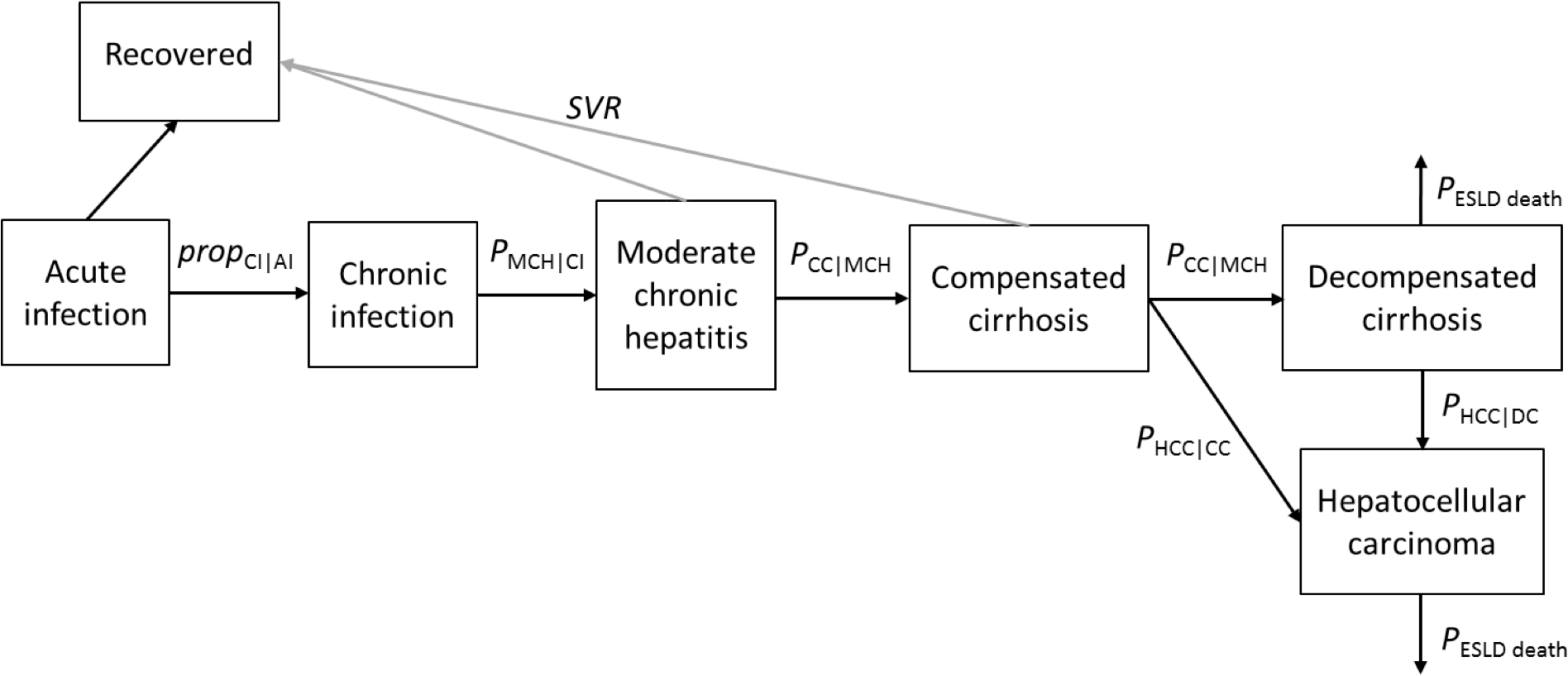
The compartmental HCV disease progression model.

The natural history of HCV disease—from acute infection through severe sequelae and death—was simulated using a compartmental model comprising eight health states (Fig 3). Model parameters, namely the proportion of acute infected patients that develop chronic infection (typically after 6 months), the annual transition probabilities between the various disease states, and excess mortality due to non-liver related causes are taken from the literature (Table 1). Several of these transition probabilities are age-specific; therefore an age-structured Markov model was developed.

**Table 1:** Progression probabilities for transitions between disease progression model states, and corresponding prior distributions.

Parameter	Probability (95% CI)	Prior distrib.	Source
*Proportions*			
Acute infected (AI) who develop chronic infection (CI)	0.74 (0.71–0.78)	*Beta*(446,157)	[49]
Acute infected (AI) who recover	1—*prop*(CI|AI)		
*Annual transitions*			
CI to moderate fibrosis (MF)	0.025 (0.018–0.033)	*Beta*(41.6,1622)	[50]
MF to compensated cirrhosis (CC)	0.037 (0.025–0.052)	*Beta*(27.8,722)	[50]
CC to decompensated cirrhosis (DC)	0.065 (0.040–0.092)	*Beta*(22.4,322)	[51]
CC to hepatocellular carcinoma (HCC)	0.014 (0.005–0.028)	*Beta*(5.60,394)	[52]
DC to HCC	0.014 (0.005–0.028)	*Beta*(5.60,394)	[52]
Mortality following DC	0.186 (0.137–0.250)	*Beta*(33.7,147)	[51]
Mortality following HCC	0.605 (0.545–0.676)	*Beta*(129,84.1)	[51]
*Background mortality rates*, *following all states*			
<1 years to 75+ years (5-year groups)	Ranging from 0.00023 (1–4, 5–9 yrs) to 0.00686 (75+ yrs)		2011 values from Reference [28]
*Excess non-liver related mortality (standardised mortality ratio (SMR))*			
	15–24 years	3.68 (2.31–5.57)	Age-group specific SMRs (10-year widths) from Reference [22]
	25–34	2.60 (2.72–3.88)	
	35–44	3.51 (2.99–4.08)	
	45–54	3.53 (2.94–4.21)	
	55–64	2.25 (1.72–2.89)	
	65–74	1.53 (1.18–1.94)	

Background mortality probabilities were derived from the national age-specific mortality rates for Malaysia, adjusted for the degree of excess non-liver-related mortality calculated from persons diagnosed HCV Ab+ in Scotland (1991–2005), in which the window of 6 months subsequent to HCV diagnosis date is excluded from follow-up time to reduce bias associated with testing individuals presenting with disease.

The eight disease progression states are: acute infection (AI), recovered (R), chronic infection (CI), moderate fibrosis (MF), compensated cirrhosis (CC), DC, HCC, and death from DC/HCC. MF corresponds to Ishak fibrosis stage F3-F5, and CC to Ishak stage F6 [21]. Individuals (i.e., both PWID and non-PWID) enter the model with AI (the left-most box in Fig 3) at ages determined by random sampling from the relevant age-at-infection distribution, and then progress through the various disease stages according to the pre-specified annual transition probabilities while simultaneously advancing in age. There is a risk of mortality in all states from non-liver-related causes (i.e., background mortality). Published Malaysian mortality rates, combined with standardised mortality ratios (SMRs) for non-liver-related excess mortality (15–75 years only; Table 1) from a Scottish study [22] were converted to annual mortality probabilities. The age range <1 year through 75 years was simulated, with everyone assumed to die after their 75th year.

The following outcomes were projected for each year in the simulation period: the prevalent number of chronically infected persons; incidence of CC, DC, HCC, and deaths from both DC and HCC. Incidence rates (per 100,000 population) were also calculated for 2010 based on the national Malaysian age-stratified population from the 2010 census [14].

### Computing disability-adjusted life-years (DALYs)

An established composite measure of disease burden is the disability-adjusted life-years (DALY) measure [23], [24]. The idea behind the DALY measure is that the impact of a particular disease can be divided into the number of years of life lost (i.e., premature mortality) and the number of years lived with a disease (morbidity) with respect to the ideal life expectancy, yielding a single measurement unit that quantifies the years of healthy life lost due to infection with a particular pathogen.

The DALY for a health state is the sum of two components: (a) premature mortality, quantified as the number of years of life lost (Years of Life Lost = YLL), computed as the number of deaths multiplied by the remaining life expectancy at age of death; and (b) morbidity, the number of years lived with that health outcome (Years Lived with Disability = YLD), computed as the number of prevalent cases multiplied by the disability weight for that outcome [25]. The DALY for HCV is therefore the sum of the YLL and YLD associated with all health states comprising the disease progression model, and is computed for a given year, yielding DALYs/year. We adopted the disability weights previously compiled for HCV within the Burden of Communicable Disease in Europe project [26]. The disability weights applied were: 0.17, 0.181, 0.209, 0.212 for symptomatic acute infection in persons aged <5 years, 5–14 years, 15–44 years, and 45+ years, respectively, 0.33 for compensated cirrhosis, 0.809 for DC and HCC [23], and 0.06 for chronic hepatitis and MHC [27]. Life expectancy (LE) values for all years of the simulation were set to the 2011 values available from the WHO Global Health Observatory [28]; because LE was stratified by 5-year age-group in this source, 1-year LE values were estimated through linear interpolation of the 5-year LE values.

### Scenario analysis

The primary scenario analysis was carried out to compare the impact of three different patterns of historical incidence on the disease burden, each of which could have given rise to the estimated prevalent number of HCV Ab+ persons in 2009 [6].

For each of these incidence scenarios (1, 2, and 3), two subscenarios were run to determine the number of incident cases of the severe disease outcomes and the DALYs that have been, and are predicted to be, averted due to current levels of antiviral therapy uptake and sustained viral clearance (SVR) rates. For instance, for the baseline historical incidence scenario 1, subscenario B indicates the current and future burden estimates under current treatment practice (see below). For subscenario A, we calculated all outcomes assuming that no patients had ever been or will be treated in future.

#### Subscenario A

No routine screening and no treatment. This represents the counterfactual disease burden; i.e., the burden expected if no public health actions or clinical care had been performed.

#### Subscenario B

Current practice. Routine screening and opportunistic testing only. The annual numbers screened/tested are unknown, but the annual number of patients treated can be estimated based on pegylated interferon drug sales, as only two pharmaceutical companies supply antiviral drugs for all of Malaysia. Sales figures indicated 400, 450, 470, 450, 480, 510, 480 and 485 treated patients from 2006 through 2013. For 2003 through 2005, the arbitrary values of 200, 300, and 350 were assumed. No patients were assumed to have been treated before 2003. From 2014, we assumed a treatment uptake o**f** 500 patients per year will be treated. This represents an annual treatment rate of approximately 0.1% of the chronically-infected population.

### SVR rates

Based on the characteristics and outcomes of clinical patients who received antiviral therapy at University of Malaya Medical Centre between 2008 and 2012, the genotype distribution was assumed to be 60% G3, and SVR rates 75% for G3 and 50% for G1/Other were assumed. We assumed that for 40% of the chronically infected patients who receive treatment, treatment begins during the compensated cirrhosis stage, and for the remaining 60%, treatment is initiated during the MF stage of disease progression. Patients achieving an SVR move immediately to the ‘Recovered’ compartment of the model and thus cannot advance to later stages of disease progression. With the assumed genotype distribution, treatment of 500 patients per year yields an expected 325 patients per year who achieve a SVR.

The Markov disease progression model was implemented and run using R version 3.0.3 [29]. Markov-chain Monte-Carlo (MCMC) sampling methods in a Bayesian framework were used to propagate uncertainty in transition probabilities and other parameters to the final incidence and DALY estimates. For this full Bayesian model, sampling from the posterior distributions for each parameter was carried out via MCMC simulation using OpenBUGS version 2.2.2 [30] and the BRugs package for R [31]. One thousand MCMC samples were discarded as burn-in, and the next 1000 samples per chain were taken to form the posterior distributions for all parameters

## Results

For each incidence scenario, subscenarios A and B were compared to determine the number of incident CC, DC and HCC cases averted, the number of averted deaths from either DC or HCC, and DALYs averted, in the final year of the simulation period. Table 2 summarises these results, and also provides the projected prevalence of chronic infection at two future time points (2025 and 2039). In the baseline Scenario 1-B, the forecast disease burden for the year 2039 is 94,900 DALYs/year (95% credible interval (CrI): 77,100–124,500), of which 47% of the burden is from premature mortality (YLL is 44,900; 95% CrI: 29,500–68,400). A predicted 2,002 (95% CrI: 1,340–3,040) patients will progress to DC and 540 (95% CrI: 251–1030) patients will develop HCC in that year. Compared with Scenario 1-B, an approximately 13% lower disease burden is forecast for 2039 under incidence Scenario 3-B, and slightly lower still (15%) for Scenario 2-B, in which the peak incidence of HCV infection mimicked that for HIV.

**Table 2:** Results of model runs under three different historical incidence scenarios.

Incidence scenario	Prevalence of chronic infection		Incidence / burden (in 2039)				Averted (in 2039)				
	In 2025	In 2039	DC	HCC	DALYs	YLL	CC	DC	HCC	Deaths	DALYs
1-A. None	455,500	528,200	2112	570	98,200	47,100	–	–	–	–	–
1-B. Current	451,500	523,500	2002	540	94,900	44,900	90	110	30	106	3300
2-A. None	405,500	441,600	1867	506	83,700	40,200	–	–	–	–	–
2-B. Current	401,000	437,000	1759	477	80,500	38,200	88	108	29	104	3200
3-A. None	420,900	422,800	1951	529	85,800	43,600	–	–	–	–	–
3-B. Current	416,900	418,100	1842	499	82,500	41,400	89	110	30	106	3300

(Scenario 1: exponentially rising incidence in PWID, 1960 to 2006; Scenario 2: exponentially rising from 1960 and then mimicking the timing of the peak of reported HIV cases among PWID, 1994–2006; and Scenario 3: exponentially rising incidence in PWID, with a declining incidence in both PWID and nonPWID from 2010), comparing the subscenarios in which no treatment had been/will be offered (-A), to the subscenario in which current antiviral treatment practice is extended to the end of the simulation period (-B). Point estimates only are indicated. DC = decompensated cirrhosis; CC = compensated cirrhosis; HCC = hepatocellular carcinoma; DALYs = disability-adjusted life-years

**Fig 4:**
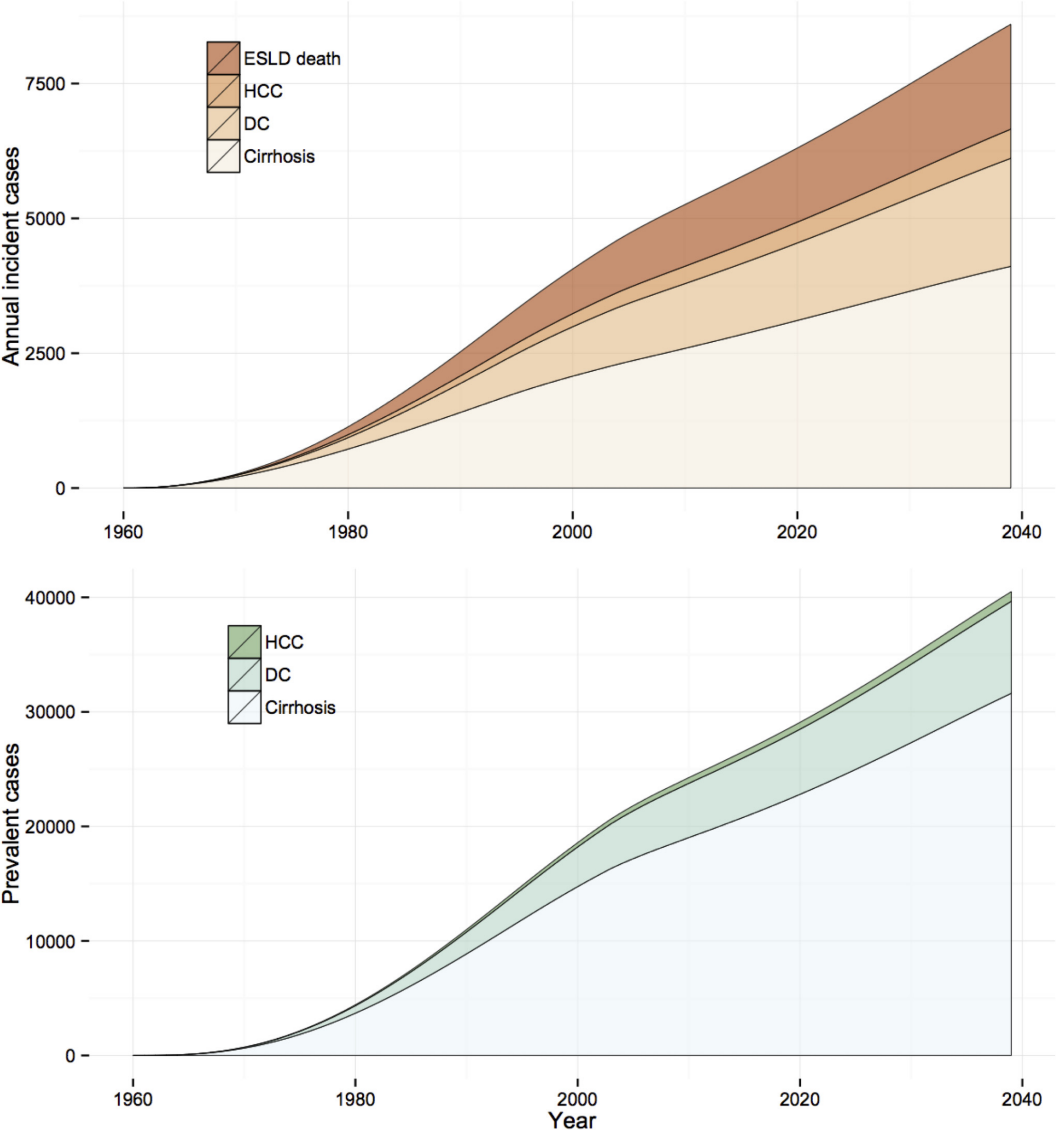
Projections of HCV-related disease outcomes for Scenario 1-B, over the period 1960–2039. Projected incidence and prevalence are shown in the upper and lower panels, respectively.

**Fig 5:**
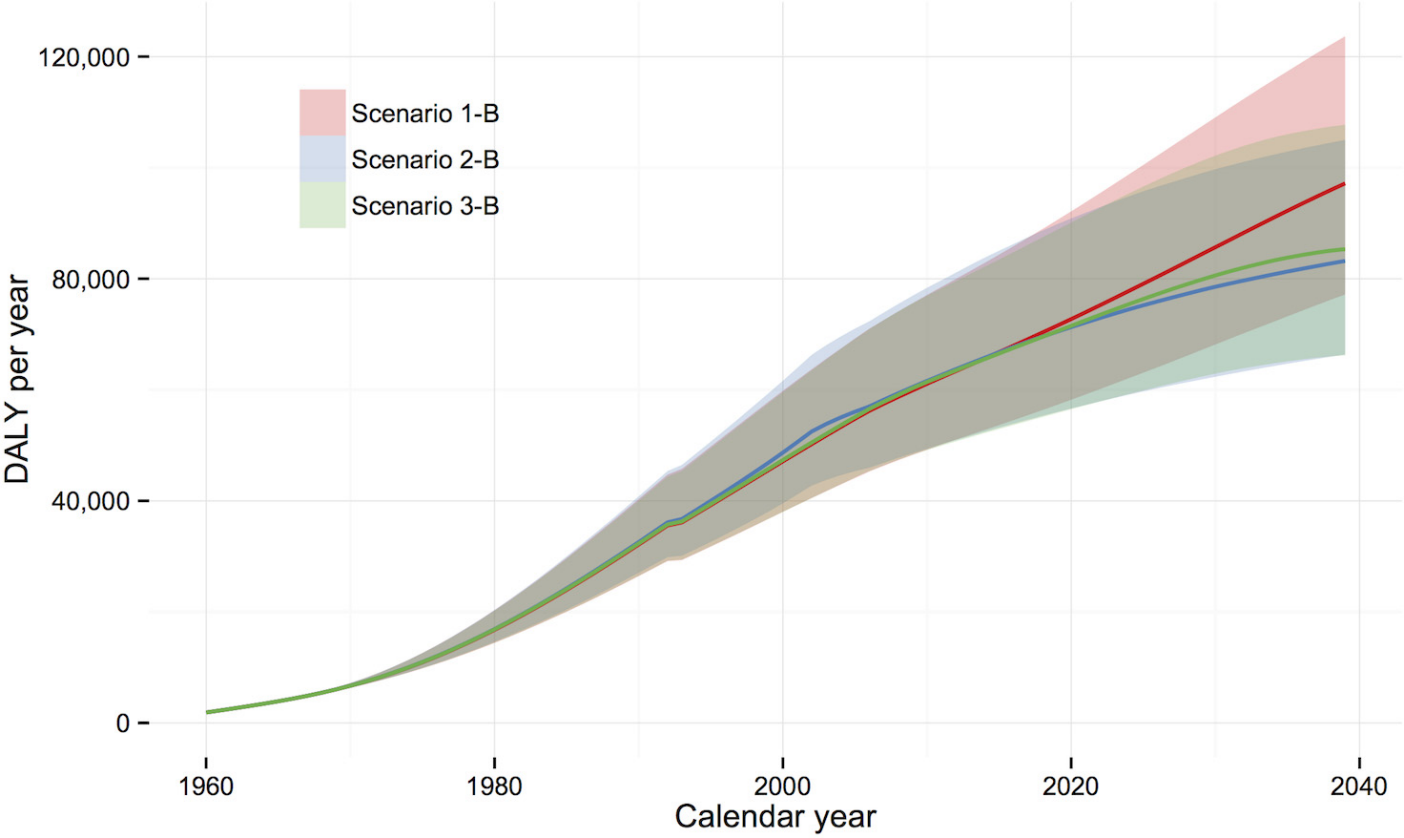
Projected HCV-related disease burden in DALYs/year, over the period 1960–2039. Three historical incidence scenarios are compared (1-B, 2-B, and 3-B), with current treatment uptake and SVR rates assumed to apply to the year 2014 onwards.

The comparison between subscenarios A and B can be considered as an estimate of the number of severe outcomes and DALY that have already been, or will be, prevented by current treatment practice. In the baseline scenario, a predicted 110, 30, and 106 new DC cases, HCC cases, and DC/HCC deaths per year, respectively, would be averted in 2039 if current treatment levels and SVR rates are maintained. Fig 4 shows the expected prevalence and incidence of the HCV disease burden indicators over the simulation period under Scenario 1-B, and Fig 5 compares, in terms of DALYs over the simulation period, the three incidence scenarios, assuming current treatment practice (i.e., Scenarios 1-B, 2-B, and 3-B). Under the assumption that current treatment levels and SVR rates are extended to 2039, the expected cumulative number of DC/HCC deaths averted by the end of the simulation period is 2,200 for Scenario 1; this represents 3.4% of the total projected number of DC/HCC deaths by 2039 (*n* = 63,900).

Model projections for the year 2010 yielded incidence rates of 4.3 (95% CrI: 2.8–6.3), 1.1 (95% CrI: 0.5–2.2), and 4.1 (95% CrI: 2.7–5.9) per 100,000 population for DC, HCC, and DC/HCC deaths, respectively.

**Fig 6:**
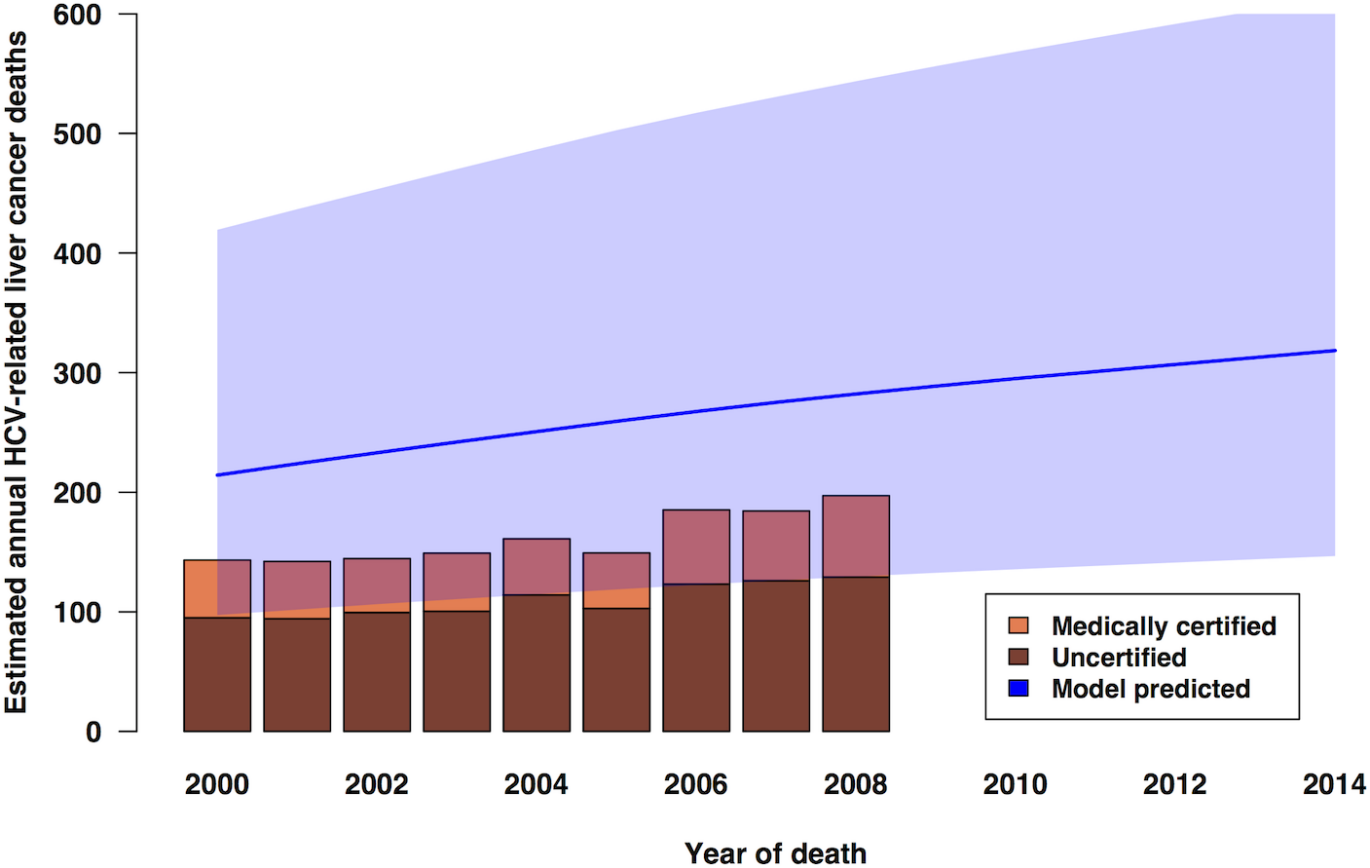
Calibration of model predictions for hepatitis C-related HCC deaths. Predictions (1995–2009; line) with 95% credible interval (shaded area) under Scenario 1-B are compared with estimated HCV-attributable liver cancer deaths plotted as bars (data for 2000–2008 only were available).

In Fig 6, model calibration is roughly assessed by plotting the model-predicted deaths from HCC together with the nationally registered deaths from liver cancer (ICD-10 code C22) in the period 2000 to 2008 [32], [33], [34], adjusted for an aetiological fraction of 18% for HCV [35]. The model predictions for the annual number of deaths from HCC are approximately 55% higher than the estimated annual number of notified (both medically certified and uncertified) deaths, but there is considerable uncertainty around the projected number of HCV-related HCC deaths (see Fig 6).

## Discussion

Given assumptions about the size, age distribution, and rate of increase of the HCV-infected population, and progression probabilities between HCV disease stages, we estimated the current disease burden, and forward-projected the future burden, attributable to HCV infection under three historical incidence scenarios. This study offers the first projections of the burden of HCV in Malaysia. We emphasise that few actual data were available to constrain the assumptions underlying the historical incidence time/series (which we have addressed by exploring several plausible scenarios); consequently, the current projections may change if new data become available. Further, the uncertainty associated with all outcome projections must be considered when interpreting the results.

A high HCV disease burden was forecast for 2039—with some variation between historical incidence scenarios—with a predicted 2,002 and 540 new DC cases and new HCC cases, respectively, occurring in 2039 under the baseline scenario 1-B. Relatively high numbers of incident infections per year, together with plausible assumptions regarding the rates of progression to severe sequelae of chronic infection and very low treatment rates, must give rise to a high future disease burden. We estimated a median burden of 94,900 (95% CrI: 77,100–124,500) DALYs in 2039 if the numbers of patients initiated on antiviral therapy and SVR rates continue at current levels. Although 90 new cirrhosis cases and 106 DC/HCC deaths are predicted to be averted in 2039—in comparison with the counterfactual no-treatment situation—treatment initiation rates at current levels are clearly insufficient to control the epidemic. The assumed annual number of new chronically-infected patients is an order of magnitude greater than the expected number of patients who are successfully treated: under Scenario 1-B, approximately 9,452 and 5,310 new chronic infections in PWID and non-PWID risk groups, respectively, are projected to occur in 2015.

Projections under the three historical incidence scenarios were broadly similar with respect to projected outcomes, with the incidence pattern mainly influencing the timing of the associated burden. Under Scenario 2, where incidence among PWID was assumed to follow the approximate shape of the HIV epidemic, approximately 15% fewer DALYs and fewer new DC and HCC cases were projected for 2039, compared with Scenario 1. As expected, the decline in AI incidence simulated from 2010 in Scenario 3 did not have an appreciable impact on burden within the relatively short remaining simulation period. However, the growth in size of the chronically infected population slowed, with only an additional 1,200 living chronic patients forecast between 2025 and 2039 (Table 2).

From the evidence synthesis estimate of the number of prevalent living HCV Ab+ individuals in 2009 [6], 59% of all Ab+ cases (males only: 66%) were estimated to have acquired their infection through injecting drug use. The remaining infections must therefore have been acquired via other transmission modes (e.g., blood products, haemodialysis, non-sterile medical injection, occupational exposure, tattooing, sexual transmission, perinatal, etc), and presumably occur amongst diverse segments of the population (i.e., not primarily amongst those individuals considered ‘high-risk’: for example, drug users, prisoners, sex workers). This finding—that 41% of prevalent cases are not among active or ex-PWID—has important implications for screening policy.

The current number of patients receiving antiviral therapy in Malaysia is very low. Even if the annual number of patients initiated on treatment is doubled, to 1000 patients per year, this still only represents a treatment uptake rate of 0.30% (3.0 per 1000 chronically-infected patients). Much higher rates of treatment uptake—even in the PWID setting—are currently being achieved, for instance in Australia, where in 2009–2010 uptake was 1.5%–2.0%, or 15 to 20 per 1000 infected [36]. Although there is no data available for the Malaysian setting, treatment with pegylated interferon and ribavirin has been found to be cost-effective in other countries [37],[38],[39] particularly in G3 patients who are the majority of infected individuals in Malaysia. New direct-acting antiviral (DAA) therapies have recently become available with better SVR rates [40] but these new agents come with high prices [41], and some may not be cost-effective in G3 patients compared with standard care [42],[43]. An economic evaluation assessing the cost-effectiveness of different treatment strategies within the Malaysian setting would be timely and beneficial for guiding management and treatment policy for chronically HCV-infected patients.

Our model-based projection study has a number of strengths and weaknesses. Strengths are the use of previously derived Ab+ prevalence estimates to constrain the incidence time-series, and the testing of three different historical incidence scenarios. The converging future disease burden estimates indicate that outcomes are not overly sensitive to the historical incidence time-series assumed.

The principal limitation is a lack of data on incidence trends, for both the distant past and the most recent period. Because of the long natural history of the disease, only a small proportion of patients who became chronically infected in the year 2015 would progress to cirrhosis and subsequent disease states by 2039. Although one would not see the impact of incorrect assumptions regarding recent incidence until 20–30 years in the future, projections further ahead in time could be markedly under- or over-estimated [44]. This limitation is shared with many HCV disease progression models, and all models fail in the accurate prediction of future incidence trends. In two of the scenarios investigated, we assumed a stable annual number of new infections among PWID from 2006 onwards (coinciding with the deployment of harm reduction measures), but incidence may rise or decline in future. Such an abrupt impact of harm reduction initiatives is a clear simplification, as the impact on is more likely to have been gradual, and may have been apparent even before the roll-out of the national programme [16]. In addition, due to lack of data we adopted the assumption of a historical linear rise in incidence among non-PWID in line with national population growth trends, which is certainly an over-simplification. Studies to collect information on HCV incidence in PWID and in incarcerated individuals in Malaysia are currently being planned.

The current model employs annual transition probabilities derived from HCV monoinfected populations; these parameters lead to a predicted 6.5% of chronically-infected patients developing cirrhosis with 20 years. Given that fibrosis progression is more rapid in HCV/HIV co-infected persons [45], and a significant proportion of Malaysia´s PWID are co-infected with HV [6], we roughly estimated the incidence of severe outcomes in 2039 after dividing HCV-infected PWID into subpopulations of HCV monoinfected and HCV/HIV co-infected PWID, specifying a faster rate of fibrosis progression for the latter. Using the estimate that 35% of HCV-infected PWID are HIV co-infected [6], and assuming a rate ratio of 2.0 for the rate of progression to cirrhosis in co-infected compared with mono-infected persons [45], we adjusted the annual transition probabilities of CI leading to MF and MF leading to CC accordingly to achieve this doubling in the rate of cirrhosis development. This allowed estimation of the extra incident CC, DC, HCC cases, and DC/HCC deaths expected in 2039. Given these assumptions, incidence in 2039 would be 12–15% higher than currently projected under the ‘monoinfected-only’ model. However, incorporation of excess mortality associated with HIV infection may change these estimates.

The discrepancy between the estimated notified deaths from HCV-related liver cancer, and the higher model-predicted annual HCC deaths (Fig 6), remains to be clarified. Either the registered deaths under-report the true number of liver cancer deaths, or the aetiological fraction for HCV is unrealistically low, or the model over-predicts HCC mortality.

We have not modelled the potential benefits of treatment as prevention; treatment of active PWID can reduce onward transmission of the virus and thus larger benefits from higher annual numbers of treated patients would be expected [46]. Modelling studies have shown that given a minimum HCV prevalence level in the PWID population, treating active PWID can be cost-effective [47]. However, given the current very low treatment numbers, incorporating transmission dynamics into the model would provide little added insight.

Finally, rates of progression between disease states are assumed to be constant when in fact they may be non-linear, and may vary with age where we have assumed age-independent rates. This is a limitation shared with other HCV natural history models, which can only be rectified with better clinical data on disease progression.

Neither the ‘no screening or treatment’ nor the ‘current treatment uptake’ subscenarios were meant to represent realistic public health/clinical care options. These were simulated only to evaluate the impact on the projected disease burden from the introduction of current treatment practice and extension of current annual numbers of patients achieving an SVR into the medium-term future. However, given the imminent availability of effective direct-acting antiviral therapies and expected increase in public health spending aimed at addressing HCV, higher SVR rates and higher levels of treatment uptake—with a consequent reduction in the present forecast disease burden—are anticipated. These DAAs, if proven cost-effective within the Malaysian setting, may change the picture dramatically [48].

In summary, the prevalence of cirrhosis and the incidence of end-stage liver disease and death due to chronic HCV infection is projected to rise steeply over the next 25 years. A detailed economic analysis is required to estimate the implications of these burden projections for future public health expenditure associated with HCV infection. Increasing HCV screening and treatment rates, together with intensive efforts to reduce transmission (e.g., through stepped-up harm reduction, education, and other prevention initiatives), would appear essential to address the high predicted HCV disease burden in Malaysia.
